# Combined TyG index and systemic inflammation indices as predictors for asteroid hyalosis: a hospital-based cross-sectional study

**DOI:** 10.3389/fendo.2026.1804058

**Published:** 2026-08-17

**Authors:** Hanying Wang, Wei Wang, Peipei Jia, Pengfei Shi, Jianan Liu, Xiaolu Cao

**Affiliations:** Hebei Ophthalmology Key Lab, Hebei Eye Hospital, No. 399, Quanbei East Street, Xingtai, Hebei, China

**Keywords:** asteroid hyalosis, cerebral infarction, diabetic retinopathy (DR), SII index, TyG

## Abstract

**Purpose:**

This research explored the laboratory indicators and derived indicators of patients with asteroid hyalosis (AH) in patients with diabetic retinopathy (DR), calculated the TyG index and systemic immune-inflammatory (SII) index, so as to identify the systemic pathogenic factors of AH in DR patients.

**Methods:**

The data were gained from patients who underwent vitrectomy for proliferative DR (PDR) in Hebei Eye Hospital. Depending on the presence or absence of AH, these participants were classified into the AH group (n = 45; 27 males, 18 females) and the non-AH group (n = 90; 48 males, 42 females). The laboratory indicators, TyG index, and SII index were compared between the two groups. A statistical analysis was implemented on their age, gender, laboratory indicators, TyG index, and SII index.

**Results:**

The patients in the AH group exhibited remarkably higher cholesterol (4.85 *vs*. 4.09 mmol/L), low-density lipoprotein (2.67 *vs*. 2.23 mmol/L), triglyceride (1.75 *vs*. 1.31 mmol/L), TyG index (9.14 *vs*. 8.77), and fasting blood glucose (6.95 *vs*. 5.82 mmol/L) levels than the patients of the non-AH group (all *P* < 0.05); also, the SII index (524.62 *vs*. 413.66), neutrophils (4.03 *vs*. 3.45 × 10^9/L), and platelets (256.00 *vs*. 222.00 × 10 ^ 9/L) significantly differed between groups (*P* < 0.05). However, the two groups displayed no statistically significant difference in other indicators, including white blood cells, monocytes, and glycosylated hemoglobin (*P* < 0.05).

**Conclusion:**

The abnormal lipid metabolism, inflammatory activation, and poor glycemic control in patients with AH are more pronounced than in DR patients without AH. Cerebral infarction and AH share overlapping systemic pathological mechanisms.

## 1 Introduction

Asteroid hyalosis (AH) is an ophthalmic disease related to changes in retinal vascular permeability and increased exudation of lipid compositions. AH is a benign degenerative disease characterized by crystalline deposits composed of calcium soaps, and most cases are unilateral lesions, usually asymptomatic, which will not cause vision loss (1). AH is more common in elderly patients, and some patients are often accompanied by systemic abnormalities such as diabetes, cerebral infarction, hypertension, etc. Studies have reported systemic serum total cholesterol (TC) and triglyceride (TG) levels in AH patients and proposed that the onset of AH is correlated with the increase of serum TG levels, while the onset of AH in non-diabetic patients is linked to the increase of TC levels.

The triglyceride-glucose (TyG) index is a composite index calculated by fasting TG [mg/dl] × fasting blood glucose (FBG) [mg/dl]/2, which is a research hotspot of metabolic diseases in recent years. This index can comprehensively reflect the blood glucose and TG metabolism levels of patients. Studies have revealed that TyG is linked to a variety of diseases, including diabetes. Additionally, the higher TyG index is associated with a higher prevalence of cardiovascular diseases, and it is a risk factor for atherosclerotic cardiovascular diseases. The increase of the TyG index raises the risk of myocardial infarction (MI), stroke, revascularization, cardiovascular death, and all-cause mortality, and it can be affected by many factors, such as hyperlipidemia and diabetes (2); lipids are an important component of vitreous asteroid bodies, and their formation may be closely related to serum cholesterol levels and TG levels. However, there is currently no research providing direct evidence for the association between the TyG index and AH.

The systemic immune-inflammatory (SII) index is a new and reliable indicator that can comprehensively assess the systemic immune and inflammatory levels of subjects by multiplying the platelet count by the neutrophil count divided by the lymphocyte count. It has been revealed that the rise of SII may be associated with an increased prevalence of diabetes, and SII may be a predictive indicator for diseases (3). Immune response participates in the process of vitreous degeneration, and immune-inflammatory response exerts a vital role in vitreous degeneration, involving multiple pathways, such as immune cell infiltration, activation of the complement system, cytokine imbalance, matrix protein immune response, and oxidative stress. Many of these pathways involve in promoting the progression of AH, as a type of vitreous degeneration.

The surgery of patients with diabetic retinopathy (DR) complicated with AH is quite difficult with poor efficacy; metabolic indicators such as TG and glucose, as well as inflammatory indicators such as TyG and SII, can better reflect the systemic inflammatory status, glucose and TG metabolism, thus better predicting the occurrence of AH in diabetic patients. Intervention of such indicators in advance can control the progression of AH in diabetic patients and provide a better evidence-based basis for visual prognosis.

## 2 Materials and methods

### 2.1 Baseline characteristics

Patient data of proliferative DR (PDR) treated with vitrectomy were collected from Hebei Eye Hospital, which covers patient populations across northern China. These participants were classified into the AH group (n = 45; 27 males, 18 females) and the non-AH group (n = 90; 48 males, 42 females) depending on the presence/absence of AH. By consulting the electronic medical record system, this research retrospectively collected the baseline clinical data of patients, encompassing age, gender, height, weight, history of diabetes, history of hypertension, and history of cerebral infarction. The results of fasting blood samples were recorded within 24 hours of admission, and the measured blood biochemical indicators [white blood cell (WBC) count, TC, low-density lipoprotein (LDL), serum creatinine (SCr), FBG, glycosylated hemoglobin (HbA1c), TG, and TyG index] were recorded. Venous blood samples of all subjects were collected after an overnight fast of at least 8 hours. Fasting triglyceride (TG) and fasting blood glucose (FBG) levels were measured using an AU5800 Series Chemistry Analyzers (Beckman Coulter, USA). Peripheral blood routine indicators including neutrophils, lymphocytes and platelets were detected by an XN-550 Blood Cell Analyzer (SYSMEX, Japan).Calculation formula for TyG index: [fasting TG (mg/dL) × FBG (mg/dL)/2]; SII = (neutrophil count × platelet count)/lymphocyte count;

All diabetic participants received standardized glycemic management at Hebei Eye Hospital, including regular hypoglycemic treatment with oral antidiabetic drugs, insulin monotherapy or combined insulin regimens according to individual blood glucose status. Routine fasting blood glucose and glycated hemoglobin monitoring were performed for all patients before ophthalmic examination. Detailed records of diabetes medication history were collected and documented as baseline clinical data.

### 2.2 Inclusion criteria

The diagnosis of asteroid hyalosis was defined as numerous small white opacities suspended within the vitreous cavity observed under slit lamp or fundus ultrasound., displaying small snowballs (the manifestation of hyperechoes) in ultrasound images. Two experienced ophthalmologists independently reviewed all ocular images; inconsistent diagnostic results were confirmed via consensus discussion.

### 2.3 Exclusion criteria

Patients with systemic long-term use of hormones for various reasons and patients with concomitant malignant tumors were excluded.

### 2.4 Statistical approaches

Statistical analyses were performed using SPSS 27.0 software. The normality of continuous variables was tested by the Shapiro–Wilk test. Normally distributed data were described as mean ± standard deviation and compared using independent sample t-tests; skewed data were presented as median (interquartile range) and compared via the Mann–Whitney U test. Categorical variables were summarized as frequencies and percentages, with intergroup differences assessed by the χ² test.

Univariate binary logistic regression was applied to screen potential risk factors for AH. Variables with P < 0.05 in univariate analysis were incorporated into multivariate logistic regression to identify independent biomarkers, with age, hypertension and diabetes duration adjusted as confounding factors. Receiver operating characteristic (ROC) curves were plotted to calculate the area under the curve (AUC), sensitivity and specificity, so as to evaluate the predictive value of TyG index and SII alone and in combination for asteroid hyalosis. P < 0.05 was considered statistically significant.

This retrospective observational cohort study was approved by the Institutional Ethics Committee (Approval No:2026LW22). A formal waiver of individual written informed consent was granted by the ethics committee due to the retrospective nature of the research and incomplete accessible contact information of partial participants. All clinical imaging and laboratory data were fully anonymized prior to statistical analysis to eliminate identifiable personal information, and all research protocols were conducted in accordance with the tenets of the Declaration of Helsinki.

## 3 Results

### 3.1 Baseline characteristics

The patient age in the AH group (60.98 ± 6.48 years) was dramatically higher than that in the non-AH group (56.71 ± 11.24 years), showing a statistically significant difference between groups (t = -2.791, *P* = 0.006). Additionally, the results showed a significantly higher proportion of AH patients with a history of cerebral infarction than that of non-AH patients with a history of cerebral infarction (24.44% *vs*. 11.11%; χ² = 4.060, *P* = 0.044). Insignificant difference was found regarding gender, coronary heart disease (CHD), smoking history, drinking history, and hypertension history between the groups (*P* > 0.05).

In terms of blood lipid and metabolic indicators, the levels of cholesterol (4.85 *vs*. 4.09 mmol/L), LDL (2.67 *vs*. 2.23 mmol/L), TG (1.75 *vs*. 1.31 mmol/L), TyG index (9.14 *vs*. 8.77), and FBG (6.95 *vs*. 5.82 mmol/L) were significantly higher in the AH group when compared with the non-AH group (all *P* < 0.05); the SII index (524.62 *vs*. 413.66), neutrophils (4.03 *vs*. 3.45 × 10^9/L), and PLT (256.00 *vs*. 222.00 × 10^9/L) also exhibited significant differences between the two groups (*P* < 0.05), suggesting that the AH group had abnormal lipid metabolism, inflammation activation, and poorer blood glucose control. There was no statistically significant difference (*P* > 0.05) in other blood indicators, such as WBC, monocytes, and HbA1c, between these two groups, as outlined in **Table 1**.

**Table 1:** A differential analysis of baseline characteristics and laboratory indicators between the two groups of patients.

Variables	T2DM-PDR (n = 90)	AH (n = 45)	Statistic	*P*
Age (year)	56.71 ± 11.24	60.98 ± 6.48	t=-2.791	0.006
WBC count (10*9/L)	6.11 (5.15, 6.92)	6.11 (5.13, 7.10)	Z=-0.560	0.575
Monocytes (10*9/L)	0.34 (0.29, 0.40)	0.32 (0.27, 0.42)	Z=-0.334	0.738
Cholesterol (mmol/L)	4.09 (3.52, 4.89)	4.85 (4.35, 5.77)	Z=-3.564	<.001
HDL (mmol/L)	0.99 (0.81, 1.11)	1.01 (0.89, 1.20)	Z=-1.478	0.140
LDL (mmol/L)	2.23 (1.78, 2.73)	2.67 (2.03, 3.43)	Z=-2.469	0.014
HbA1c (%)	7.53 (6.71, 8.64)	7.80 (6.90, 9.20)	Z=-0.922	0.357
BUN (mmol/L)	5.87 (4.77, 7.45)	5.51 (4.84, 8.34)	Z=-0.250	0.803
Uric acid (umol/L)	316.50 (262.25, 363.50)	313.00 (269.00, 386.00)	Z=-0.138	0.890
SCr (umol/L)	63.00 (55.25, 73.75)	66.00 (52.00, 93.00)	Z=-1.116	0.264
TBIL (umol/L)	8.95 (6.40, 10.88)	8.85 (6.50, 11.90)	Z=-0.413	0.679
DBIL (umol/L)	1.80 (1.30, 2.30)	1.80 (1.40, 2.30)	Z=-0.107	0.914
PLT (10*9/L)	222.00 (184.50, 265.00)	256.00 (214.00, 278.00)	Z=-1.986	0.047
Neutrophils (10*9/L)	3.45 (2.80, 4.06)	4.03 (2.90, 4.80)	Z=-2.084	0.037
Lymphocytes (10*9/L)	1.97 (1.61, 2.33)	1.82 (1.34, 2.17)	Z=-1.678	0.093
SII	413.66 (268.86, 543.65)	524.62 (352.65, 772.38)	Z=-2.747	0.006
TG (mmol/L)	1.31 (0.99, 1.94)	1.75 (1.31, 2.62)	Z=-3.349	<.001
FBG (mmol/L)	5.82 (4.86, 7.16)	6.95 (5.56, 8.62)	Z=-2.488	0.013
TyG index	8.77 (8.35, 9.18)	9.14 (8.87, 9.61)	Z=-3.722	<.001
DBP	78.00 (71.00, 86.00)	82.00 (75.00, 85.00)	Z=-0.642	0.521
SBP	132.00 (118.00, 142.00)	135.00 (123.00, 143.00)	Z=-0.511	0.609
Course of diabetes	15.00 (8.25, 20.00)	18.00 (8.00, 20.00)	Z=-0.707	0.480
Gender, n(%)			χ²=0.540	0.462
Male	48 (53.33)	27 (60.00)		
Female	42 (46.67)	18 (40.00)		
Cerebral infarction, n(%)			χ²=4.060	0.044
No	80 (88.89)	34 (75.56)		
Yes	10 (11.11)	11 (24.44)		
Smoking history, n(%)			χ²=0.335	0.563
No	68 (75.56)	36 (80.00)		
Yes	22 (24.44)	9 (20.00)		
Drinking history, n(%)			χ²=3.086	0.079
No	74 (82.22)	31 (68.89)		
Yes	16 (17.78)	14 (31.11)		
Hypertension, n(%)			χ²=1.215	0.270
No	43 (47.78)	17 (37.78)		
Yes	47 (52.22)	28 (62.22)		
CHD, n(%)			χ²=0.109	0.742
No	76 (84.44)	37 (82.22)		
Yes	14 (15.56)	8 (17.78)		

**Table 1** summarizes the demographic, clinical and laboratory baseline indicators of subjects stratified by the presence or absence of asteroid hyalosis. Patients with asteroid hyalosis exhibited significantly higher TyG index and SII levels than the control group (all P < 0.001), whereas age and gender showed no significant intergroup differences (P > 0.05). Continuous variables with skewed distribution were presented as median (interquartile range) and compared by Mann–Whitney U test; categorical variables were analyzed via χ² test.

### 3.2 Multi-variable binary logistic regression analysis

Multivariate binary logistic regression revealed that the TyG index, rescaled SII and cerebral infarction history were independently associated with elevated odds of AH. Per one-unit increment in the TyG index, the adjusted odds of AH increased substantially (OR = 2.371, 95%CI: 1.176–4.782). Given the 33% prevalence of AH in our surgical cohort, this odds ratio cannot be directly interpreted as relative risk; model-adjusted predicted probabilities were further calculated to quantify absolute risk differences across varying TyG levels. For clinical interpretability, SII was rescaled to a per-100-unit increment, with each 100-unit elevation correlated with higher odds of AH (OR ≈ 1.49). Patients with a history of cerebral infarction exhibited markedly higher odds of AH relative to those without cerebral (**Table 2**).

**Table 2:** Multi-variable binary logistic regression analysis.

Variables	*B*	S.E	Wald	*P*	OR	95%CI	
						Lower limit	Upper limit
Age	0.043	0.026	2.695	0.101	1.043	0.992	1.098
Cholesterol	0.130	0.323	0.161	0.688	1.139	0.604	2.145
LDL	0.711	0.389	3.341	0.068	2.035	0.950	4.361
SII(per 100-unit increase)	0.400	0.001	14.407	<0.001	1.492	1.221	1.820
TyG index	0.863	0.358	5.819	0.016	2.371	1.176	4.782
Cerebral infarction	2.281	0.655	12.136	<0.001	9.788	2.712	35.327
Constant	-15.995	3.756	18.136	<0.001			

**Table 2** shows multivariate binary logistic regression analysis for asteroid hyalosis. The table reports regression coefficient (B), standard error (S.E), Wald statistic, P value, odds ratio (OR), and the lower and upper limits of the 95%CI for each candidate biomarker. SII was rescaled to a per-100-unit increment for clinical interpretability; the original OR = 1.004 per single SII unit is omitted. Odds ratios (OR) only reflect association with odds of asteroid hyalosis, not direct relative risk, given the 33% prevalence of AH in this surgical cohort.

### 3.3 The predictive value of SII, the TyG index, cerebral infarction, and their combination for AH

Receiver operating characteristic (ROC) analyses were performed to quantify the discriminative performance of SII, TyG index, cerebral infarction history, and their composite panel for predicting asteroid hyalosis (AH), with all curves generated via MedCalc 20.010’s unified nonparametric Mann-Whitney U algorithm. After recalculation to resolve the statistical inconsistency of cerebral infarction’s diagnostic metrics, updated predictive parameters are presented as follows:

The SII exhibited an AUC of 0.645 (95% CI: 0.541–0.750), with an optimal cutoff value of 507.26, yielding a sensitivity of 53.33%, specificity of 73.33%, and Youden’s J index of 0.267. The TyG index, the most robust single biomarker, achieved an AUC of 0.697 (95% CI: 0.607–0.787) at a cutoff of 8.665, accompanied by a sensitivity of 88.89%, specificity of 47.78%, and Youden’s J index of 0.367. For cerebral infarction history, the revised AUC was 0.592 (95% CI: 0.510–0.674) with a sensitivity of 24.44%, specificity of 88.89%, and Youden’s J index of 0.133; the updated 95% CI no longer crosses 0.5, consistent with its statistically significant yet weak standalone discriminative power. The combined predictive model integrating SII, TyG index and cerebral infarction history produced an AUC of 0.811 (95% CI: 0.736–0.885) at a cutoff threshold of 0.511, with a sensitivity of 71.11%, specificity of 80.00%, and Youden’s J index of 0.511.

**Figure 1:**
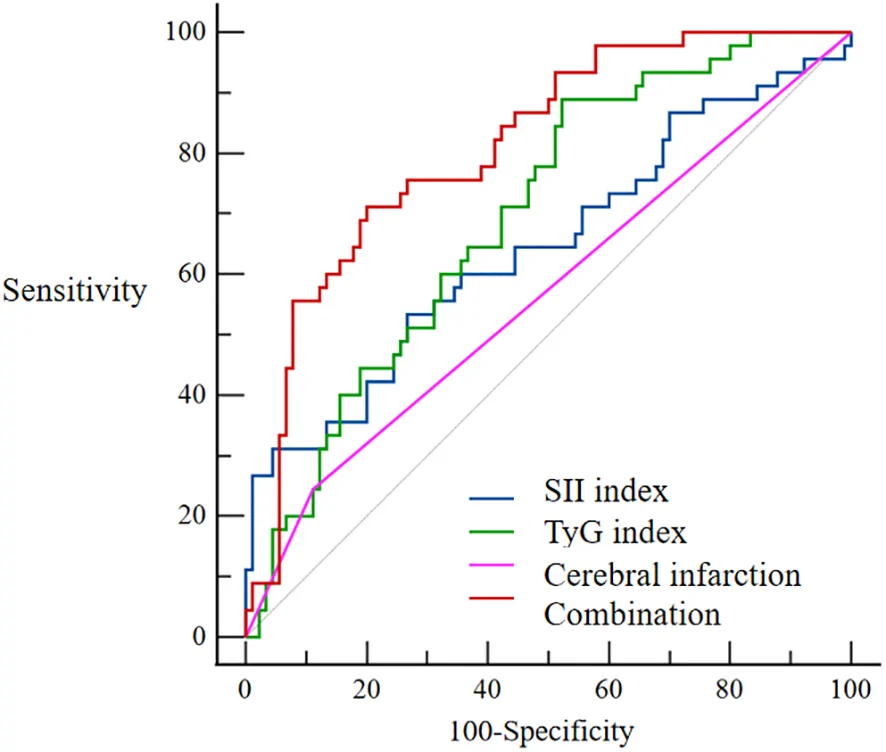
ROC curves.

Collectively, SII, TyG index and cerebral infarction history each possessed statistically significant predictive capacity for AH. The composite model yielded a markedly higher AUC (0.811), along with improved balanced sensitivity, specificity and Youden’s J index relative to every individual biomarker. Pairwise within-software ROC comparisons further verified that the multi-indicator panel achieved significantly superior discriminative efficacy over the TyG index alone, confirming that integrating multiple clinical and laboratory markers substantially enhances diagnostic performance for AH. Full diagnostic statistics are summarized in **Table 3** and visualized in **Figure 1**. Among all single predictors, the TyG index delivered moderate discriminative ability to screen patients at risk of AH. **Table 3** quantifies and contrasts the diagnostic performance of all candidate predictors, offering quantitative statistical evidence to support their potential clinical utility as predictive biomarkers for AH.

**Table 3:** Analysis of the predictive value of all independent predictors.

Variable	AUC	95%CI	Cutoff value	Sensitivity (%)	Specificity (%)	Youden index	*P*	P-value (vs TyG index)
SII (per raw value)	0.645	0.541 ~ 0.750	507.26	53.33	73.33	0.267	0.007	0.029
TyG index	0.697	0.607 ~ 0.787	8.665	88.89	47.78	0.367	<0.001	Reference
Cerebral infarction	0.592	0.510–0.674	—	24.44	88.89	0.133	0.038	<0.001
Combination	0.811	0.736 ~ 0.885	0.511	71.11	80.00	0.511	<0.001	<0.001

**Table 3** presents the predictive performance of all independent predictors screened via multivariate logistic regression for AH. The area under the receiver operating characteristic curve (AUC), optimal cutoff value, sensitivity, specificity, positive predictive value, and negative predictive value of each independent biomarker (TyG index and SII) were calculated and summarized.

**Figure 1** displays the ROC curves of TyG index, cerebral infarction, SII and their combination for predicting asteroid hyalosis. The combined model yielded the largest AUC value, with higher sensitivity and specificity than single indicators alone, indicating superior predictive ability of combined metabolic-inflammatory biomarkers. The optimal cut-off values of TyG index, cerebral infarction and SII were also labeled on the curves for clinical reference.

## 4 Discussion

Asteroid hyalosis (AH) is a common idiopathic vitreous disorder, named for the calcium- and lipid-containing deposits within the vitreous structure, the etiology of which is not fully clarified yet (1). Since it generally does not affect vision, it generally does not require special treatment. However, AH may occur simultaneously with other eye diseases, among which DR is the most common; existing studies have detailedly described these particles from both molecular and structural perspectives (4). The pathogenesis of AH has not been fully elucidated, but clinical data have shown that age is a vital relevant factor in the occurrence of AH, and the detection rate of AH gradually increases with the increase of age. In addition to age, other previously proposed indicators, including male gender, hypertension, serum TG, serum LDL, hypercalcemia, atherosclerotic cardiovascular disease, diabetes, high body weight, and drinking history, can accelerate the initiation of AH (5). The aforementioned results suggest a certain relationship between AH and metabolic diseases. The subsequent analysis revealed that systemic metabolic disorders, such as lipid metabolism abnormalities and diabetes, may become a potential risk factor for AH by affecting ocular microcirculation, promoting the abnormal deposition of lipid components in the vitreous body, accelerating vitreous liquefaction, and even vitreous degeneration. The aforesaid findings not only offer a key entry point for further exploration on the pathophysiological mechanisms of AH but also lay a theoretical foundation for risk assessment, early identification, and targeted intervention strategies for AH in clinical practice.

As a metabolic disease, diabetes has a rising incidence year by year all over the world. Its ocular complications, namely diabetic retinopathy (DR), are the main challenge in ophthalmic diseases, seriously threatening the vision and quality of life of patients. PDR even requires vitrectomy to control disease progression, and AH can coexist with DR. A comparative analysis on clinical data of DR patients with and without AH can provide more theoretical basis for studies on AH (4).

Although previous epidemiological studies have shown a certain linkage between AH and systemic diseases, particularly cardiovascular or metabolic diseases, there are few studies on AH complicated with DR. Currently, the relations between AH and the TyG index, SII index, and cerebral infarction identified in this study are rarely reported. Prior studies mainly described the correlations of AH with age, hypertension, and diabetes. It has been demonstrated that AH is associated older age, thicker lenses, and higher spherical equivalent values, but not with diabetes or other systemic indicators (6). As early as 1991, several authors reported the univariate correlations of AH with diabetes, arterial hypertension, and atherosclerosis (7). However, Fawzi et al. investigated AH in autopsy patients and found no association between AH and systemic complications through a multivariate analysis (8); Consistent with the conclusions of most previous studies, this research also found that advanced age is a risk factor for the onset of AH based on a comparative analysis of the age between the two groups; as age increases, the vitreous undergoes degenerative changes, and pathological changes occur from both matrix and ion perspectives of the vitreous body. For matrix changes, type II collagen fibers in the vitreous undergo conformational changes under oxidative damage or enzymatic hydrolysis, forming abnormally aggregated collagen fiber bundles through intermolecular cross-linking (9, 10). The exposed carboxylate groups on the surface of denatured collagen yield a sustained negative electric field, which forms a granular mixture through electrostatic adsorption of substances such as calcium and phosphorus, providing favorable conditions for the formation of asteroid bodies. Additionally, ion channel imbalance leads to abnormal metabolism of calcium and phosphorus ions (11), and abnormal deposition of denatured collagen fiber fragments, hyaluronic acid degradation products, and apoptotic cell fragments in the matrix microenvironment can all lead to the onset of AH (12).

TyG index is a composite index calculated by fasting TG [mg/dl] × FBG [mg/dl]/2, which can evaluate the glucose metabolism and insulin resistance of diabetic patients; and TyG index is associated with adverse clinical outcomes such as acute coronary syndrome, heart failure, and ischemic stroke. The TyG index is strongly linked to diabetes, CHD, and other vascular diseases and is usually applied for predicting the risk of death and adverse cardiovascular and cerebrovascular events. Growing evidence has validated the close link between insulin resistance represented by the TyG index and ocular degenerative lesions in diabetic populations. This research documented a significantly higher TyG index of the patients in the AH group than in the non-AH group. Meanwhile, studies have proven that the onset of AH is associated with changes in retinal vascular permeability and lipid metabolism (11, 13), which is coincident with our research results. The TyG index proves to be a reliable predictor of both the onset and severity of DR, with the risk of DR lesions following a U-shaped (14). The TyG index can be used as a potential key and practical indicator for DR (15). The median level of advanced glycation end products (AGEs) in the vitreous body is markedly higher in PDR patients with uncontrolled blood glucose as compared to patients with controlled blood glucose. The levels of AGEs and D-dimer in the vitreous body may reflect pathological changes in the retina, and blood HbA1c levels can be applied to evaluate the level of AGEs in the vitreous body of PDR patients. AGEs can disrupt the normal structure of collagen fibers, leading to increased degradation of hyaluronic acid and ultimately accelerating vitreous liquefaction (16); the reason is that the content of palmitate and other free fatty acids in retinal blood vessels increases with the development of diabetes. The abnormal accumulation of fatty acids will lead to apoptosis of retinal capillary pericytes, and the permeability of retinal blood vessels will be changed. The leaked substances enter the vitreous body and bind to the substances in the vitreous matrix, forming asteroid bodies (17, 18). Sustained insulin resistance reflected by high TyG triggers systemic low-grade inflammation and disrupts the inner and outer blood-retinal barrier, allowing potential increased leakage of circulating lipid and calcium components into vitreous gel to form asteroid bodies. High permeability of retinal blood vessels accelerates the onset of AH, and the increased vascular permeability caused by metabolic abnormalities may be an important bridge connecting systemic diseases with AH (19); posterior vitreous detachment and vitreous liquefaction may offer conditions for the aggregation of asteroid bodies (20). Meanwhile, comprehensive reviews of asteroid hyalosis indicated that systemic metabolic dysfunction and chronic microvascular injury are core prerequisites for AH progression, consistent with our findings that TyG retains independent predictive value despite balanced HbA1c between groups.

Abnormal blood lipid metabolism is the core risk factor of atherosclerosis, and microvessels in the eye such as the retinal vessels and ciliary vessels will have pathological changes due to abnormal blood lipid, which will indirectly damage the vitreous body; it has been found that AH in diabetic patients and non-diabetic patients is linked to the increase of serum TG level; AH in non-diabetic patients is related to the increase of serum TC levels (21).High LDL-C will deposit beneath the vascular endothelium of the eyes, forming atherosclerotic plaques that lead to vascular lumen stenosis and reduced blood flow velocity, triggering ischemia and hypoxia of ocular tissues including vitreous body; ischemia and hypoxia can activate the oxidative stress response in the eyes and induce the release of inflammatory factors such as TNF-α and IL-6, which can damage the collagen fiber network in the vitreous body (19, 22) and accelerate the degradation of hyaluronic acid, ultimately leading to a rapid transformation of the vitreous body from a “gelatinous” state to a “liquefied” state (i.e. accelerated vitreous liquefaction); after vitreous liquefaction, the lipid components in the vitreous body are precipitated and aggregated, forming stellate opacities (23).

Recently, SII has emerged as a new and reliable indicator that can measure systemic immunity and inflammatory levels of subjects, and multiple studies have confirmed that inflammatory factor indicators are strongly linked to diabetes (3); there are studies on SII and systemic diseases such as CHD, vascular stenosis, diabetes, body fat distribution, and some cancer-associated diseases to assess disease severity and prognosis, particularly diabetes. It is revealed that the level of systemic inflammation can explain the prevalence and progression of diabetes (4). This study demonstrated that high SII was highly relevant to the occurrence of AH, and retinal vascular injury and inflammatory stimulation can lead to changes in retinal vascular permeability, promoting the leakage of calcium and lipids from retinal blood vessels, changes in lipid and calcium content in the vitreous body, the formation of asteroid bodies after abnormal deposition of calcium-lipid complexes, ultimately leading to the onset of AH. In this case, AH may be a manifestation of a secondary pathological response to eye injury (24); immune-inflammatory response exerts a crucial role in vitreous degeneration, and immune cells, including phagocytes, lymphocytes, and neutrophils, infiltrate the vitreous body. After activation, these immune cells release pro-inflammatory cytokines such as tumor necrosis factor (TNF)-α, interleukin (IL)-1β, and IL-6, activating the inflammatory cascade reaction; immunoglobulins such as IgG and IgA can deposit in the vitreous body and activate the classical complement pathway. Complement activation can lead to the deposition of complement components like C3a and C5a, which exert chemotactic effects and can attract immune cells to the site of inflammation, exacerbating the inflammatory response. Pro-inflammatory factors can motivate the production of cytokines and chemokines, resulting in immune cell infiltration and exacerbation of the inflammatory response; the inflammatory response causes degradation and structural changes of vitreous matrix proteins, affecting the stability of the vitreous matrix. The deposition of autoantibodies or antigen-antibody complexes in the vitreous matrix can trigger an immune response, which may be associated with pathological changes such as the presence of spherical particles containing calcium and lipids in the vitreous body. Besides, oxidative stress may participate in the immunopathological process of AH by affecting immune cell function and the release of inflammatory cytokines. The unilateral occurrence of asteroid hyalosis can be postulated by the contribution from various factors, e.g., local inflammation, retinal pigment epithelial degeneration, local changes in pH, vitreous collagen degeneration, and most notably, increased retinal vascular permeability altering the calcium and lipid levels of the vitreous (25).Elevated SII is accompanied by overexpressed pro-inflammatory cytokines TNF-α and IL-6. Persistent inflammatory stimulation of this kind induces irreversible structural impairment of both the inner and outer blood-retinal barrier (BRB). BRB disruption disturbs the physiological homeostasis of retinal endothelial cells and pericytes at both qualitative and quantitative levels, which may thereby lead to potential increased leakage of circulating calcium and lipid moieties into the vitreous. Within the vitreous matrix, lipid-calcium complexes gradually aggregate and precipitate into asteroid bodies, the hallmark lesions of AH (19, 26–28)..

Notably, glycated hemoglobin (HbA1c) levels were comparable between the AH group and the control group in our cohort, a phenomenon attributable to the unified standardized glycemic management protocol implemented for all diabetic patients admitted to our hospital. Consistent long-term glucose control narrowed interindividual gaps in average blood glucose levels, resulting in non-significant between-group differences in HbA1c. Nevertheless, all enrolled subjects exhibited abnormally elevated HbA1c values exceeding the clinical normal threshold, which reflected poor and unstable long-term glycemic control across the entire study population. Persistent hyperglycemia drives sustained systemic metabolic dysfunction and maintains a chronic low-grade inflammatory microenvironment; collectively, these pathological conditions disrupt intraocular microcirculation and accelerate the progression of vitreous degenerative lesions including AH. Importantly, after multivariate adjustment for confounders, the TyG index and SII still displayed remarkable intergroup disparities and retained independent predictive capacity. This finding further indicates that insulin resistance and systemic chronic low-grade inflammation, rather than long-term average blood glucose reflected by HbA1c, serve as the dominant pathogenic drivers underlying AH. While we identified significant correlations between serum indicators of insulin resistance and AH, the complete pathological cascade we propose remains merely speculative, as this study lacks direct supporting evidence including vitreous biochemical profiling, blood-retinal barrier permeability testing, and quantification of intraocular cytokines. We hypothesize that insulin resistance may aggravate systemic endothelial impairment, which in turn could augment subtle leakage across the blood-retinal barrier and promote the translocation of circulating calcium and lipid moieties into the vitreous gel, ultimately facilitating the formation of asteroid bodies. This putative leakage-mediated pathway demands additional prospective laboratory investigations to establish genuine causal links.

Additionally, our analysis identified a correlative association between cerebral infarction and AH, though this relationship does not imply a direct causal effect. Instead, cerebral infarction acts as a marker of shared systemic microangiopathy underlying AH development. The present study emphasizes that no available evidence supports a direct causal effect of cerebral infarction on the development of AH. Instead, both conditions stem from long-standing metabolic disturbances and diffuse vascular endothelial injury throughout the systemic circulation. Diabetes can simultaneously damage the microvessels in the brain and eyes. Cerebral infarction and retinal degenerative diseases are manifestations of the same microvascular disease in different organs. A nationwide representative population study in the United States, which synthesized multiple pieces of previous epidemiological evidence, explicitly identified stroke, myocardial infarction, and systemic atherosclerosis as high-frequency comorbidities of AH. The study emphasized that there is no evidence indicating that the aforementioned diseases directly induce vitreous degeneration; nevertheless, both conditions share a common origin in long-term metabolic disorders and vascular endothelial injury (4). Accumulated published studies have demonstrated that patients with ischemic stroke frequently exhibit structural abnormalities of the retinal microvasculature. Quantitative and qualitative alterations in fundus microcirculation can reliably mirror the severity of cerebral microvascular damage. Given the identical embryonic origin, analogous microvascular anatomical features and shared systemic pathogenic cascades between retinal and cerebral small vessels, cerebral infarction and asteroid hyalosis represent two distinct phenotypic manifestations of generalized systemic microangiopathy rather than a direct cause-effect relationship (29). Cerebral infarction was correlated with asteroid hyalosis in our univariate analysis; however, this association does not indicate a direct causal or predictive relationship between the two conditions. Instead, cerebral infarction serves as a clinical marker of widespread systemic microangiopathy driven by chronic metabolic disturbances, which represents a shared underlying pathological background for the development of asteroid hyalosis rather than an independent risk predictor of AH. There may be a potential association and similar pathogenic processes between AH and cardiovascular diseases. Future studies can further evaluate the differences between bilateral and unilateral AH patients and explore more specific pathophysiological mechanisms.

## 5 Conclusion

Compared with DR patients without AH, those with AH demonstrated more pronounced abnormalities in lipid metabolism, systemic inflammatory activation, and glycemic control. Notably, concurrent elevation of the TyG index and SII index suggests a synergistic interplay between insulin resistance and chronic low-grade systemic inflammation. These systemic perturbations may collectively predispose diabetic individuals to stellate vitreous lesions. Furthermore, this study identified cerebral infarction as a comorbid condition sharing pathophysiological features with AH—particularly dysregulated metabolic homeostasis, suboptimal vascular risk factor management, and accumulation of multiple cardiometabolic risk factors. Metabolic and inflammatory dysfunction likely impairs intraocular microcirculation and compromises the blood–vitreous barrier integrity, thereby promoting aberrant lipid–calcium deposition and accelerating irreversible vitreous liquefaction and structural degeneration. To our knowledge, this is the first study to: (1) establish an epidemiological link between systemic metabolic–inflammatory dysregulation and vitreous degenerative disorders; (2) explicitly report an association between AH and cerebral infarction; and (3) demonstrate overlapping and potentially interactive risk profiles—including hypertension, dyslipidemia, and diabetes-related metabolic disturbances—between AH and cerebral infarction. Importantly, the findings provide robust evidence that chronic inflammation and dyslipidemia actively contribute to both the initiation and progression of AH, thereby offering a mechanistic foundation for future etiological and pathophysiological investigations.

Several methodological limitations constrain the interpretability of the present study. For one thing, its single-center nature and limited sample size weaken the generalizability of our conclusions. Cerebral infarction represented an infrequent endpoint, and the paucity of relevant cases distorted the corresponding odds ratio estimates. For another, stratified analysis was absent to quantify individual risk factor effect sizes, hindering clear differentiation of each variable’s independent role in AH occurrence. All participants were patients with PDR scheduled for vitrectomy, representing a highly selected surgical population rather than a random sample of the general population. The AH prevalence of 33% (45/135) observed herein is substantially elevated relative to the 1%–2% prevalence reported in large general-population cohorts (30), which introduces prominent spectrum and ascertainment bias. Concentrated severe metabolic dysfunction and widespread systemic microangiopathy among PDR surgical patients may inflate the discriminative performance of serum biomarkers, limiting the generalizability of our predictive models to non-surgical diabetic patients or community-based individuals. Finally, the exact molecular pathways through which inflammation and lipid disorders drive AH remain poorly defined, requiring further experimental validation. Future prospective multicenter studies enrolling unselected diabetic populations are required to validate our predictive indicators and confirm the observed associations. Future multicenter investigations with larger cohorts and molecular testing are needed to map AH risk factors and core pathogenic cascades, supporting targeted prevention and mechanistic research of this disease.

## Data Availability

The raw data supporting the conclusions of this article will be made available by the authors, without undue reservation.
